# Apoptotic function of tumor-associated antigen RCAS1 in oral squamous cell carcinoma

**DOI:** 10.1186/1479-5876-12-112

**Published:** 2014-05-06

**Authors:** Hideaki Tanaka, Takeshi Toyoshima, Kenzo Sonoda, Ryoji Kitamura, Masaaki Sasaguri, Shintaro Kawano, Ryota Matsubara, Yuichi Goto, Seiji Nakamura

**Affiliations:** 1 Division of Maxillofacial Diagnostic and Surgical Sciences, Section of Oral and Maxillofacial Oncology, Faculty of Dental Science, Kyushu University, Maidashi 3-1-1, Higashi-ku, Fukuoka 812-8582, Japan; 2 Department of Obstetrics and Gynecology, Graduate School of Medical Sciences, Kyushu University, Fukuoka, Japan; 3 Department of Science of Physical Functions, Division of Oral and Maxillofacial Surgery, Kyushu Dental University, Kitakyushu, Japan

**Keywords:** RCAS1, Oral squamous cell carcinoma, Apoptosis, Immune surveillance, Biomarker

## Abstract

**Background:**

Receptor-binding cancer antigen expressed on SiSo cell (RCAS1) is derived from uterine adenocarcinoma and can induce apoptosis in lymphocytes, allowing tumor cells to escape from immune surveillance. RCAS1 is reportedly expressed in a membranous pattern on tumor cell or soluble one in serum of patients. The aim of this study was to investigate expression patterns of RCAS1 and the effect on apoptosis in oral squamous cell carcinoma (OSCC) cell lines.

**Methods:**

In four kinds of OSCC cell lines (HSC-2, HSC-3, SQUU-A, and SQUU-B), RCAS1 mRNAs and proteins were determined by RT-PCR and immunocytochemistry. Membranous RCAS1 was determined by flow cytometry. Culture supernatants were analyzed for detection of soluble RCAS1 by dot blotting and enzyme-linked immunosorbent assay. Apoptotic ability of RCAS1 on the erythroid leukemia cell line K562 with the putative receptor was evaluated by flow cytometry in co-culture with highly metastatic SQUU-B, with knocked-down *RCAS1* cells or in a no-cell contact condition.

**Results:**

RCAS1 mRNA and proteins were expressed in all of OSCC cell lines. Membranous pattern were expressed in all cell lines, while soluble pattern was detected in all supernatants. *RCAS1* mRNA, membranous and soluble RCAS1 were significantly seen in SQUU-B more than the other 3 cell lines (*P* < 0.05). K562 apoptosis was induced in co-culture with each of all cell lines, particularly with SQUU-B. Apoptosis was markedly reduced in co-culture with *RCAS1* knockdown cells, but was induced in co-culture without cell contract of SQUU-B.

**Conlusions:**

Our study suggests that RCAS1 has an apoptotic function via membranous/soluble expression pattern in OSCC cells. RCAS1 may thus affect tumor escape from immune surveillance in OSCC by inducing apoptosis.

## Background

Receptor-binding cancer antigen expressed on SiSo cell (RCAS1) is recognized by the monoclonal antibody 22-1-1, and was initially identified in uterine adenocarcinoma [1,2]. Its expression is reportedly correlated with clinico-pathological parameters in 15 human cancers, such as clinical stage, histological subtype, differentiation, tumor size, depth of invasion, lymphovascular space involvement, lymph node metastasis, and cytological results [3-20]. RCAS1 acts as a ligand for a putative receptor in cell lines of several human tissue types and peripheral lymphocytes, such as activated T, B, and NK cells. *In vitro,* RCAS1 inhibits growth of those activated cells and then induces their apoptosis [21]. Similarly, RCAS1 can induce apoptosis of tumor infiltrating lymphocytes (TILs) in cancer patients. This induction can lead to the escape of tumor cells from immune surveillance, thus contributing to tumor growth *in vivo*.

After proteolytic process, membranous RCAS1 is secreted to serum from tumor cells [22]. This process, also called “ectodomain shedding”, affects activity of membrane protein by altering its localization and mode of action. For RCAS1, ectodomain shedding converts membranous protein to soluble one, which greatly influences its function. Soluble RCAS1 concentrations are reported via enzyme-linked immunosorbent assay (ELISA) to be significantly higher in patients with uterine cancer than in healthy blood donors [23]. Moreover, change of RCAS1 concentration during the culture was associated with response to treatments in patients with uterine and ovarian cancers. Compared with serum from healthy blood donors, serum from patients with uterine and ovarian cancers significantly inhibited growth of erythroid leukemia cell line K562 that express the putative RCAS1 receptor by inducing apoptosis [3]. Literature review revealed that RCAS1 could be considered as a biomarker since the expression was associated with important clinicopathological parameters for patient’s management and prognosis [24]. Taken together, these data indicate that soluble RCAS1 could be a valuable biomarker for cancer, with the ability to improve insight into the disease state, to facilitate diagnosis, therapeutic strategies and disease monitoring, and to inhibit growth of receptor-expressing cells. Thus, detection of soluble RCAS1 in serum would be more useful than membranous RCAS1 as a clinical indicator, since the apoptotic ability of soluble RCAS1 can impact on the total ability of tumor cell more than that of a membranous one and blood sampling is clinically easy [22].

We previously reported that the expression of RCAS1 in oral squamous cell carcinoma (OSCC) was detected both in cytoplasm and on membrane of tumor cell in 41 of 130 cases (31.5%) by immunohistochemistry. Moreover, the percentage of TUNEL positive TILs in patients with RCAS1 positive OSCC was significantly higher than in those with RCAS1 negative OSCC [25]. Furthermore, RCAS1 expression in oral epithelial dysplasia was related to earlier conversion to oral cancer [26]. However, no studies on RCAS1 expression patterns in OSCC have been reported. Moreover, the apoptotic function of RCAS1 in OSCC is also unclear. Therefore we examined expression kinetics of RCAS1 in OSCC cell lines, to whether or not expression depended on characteristics of tumor cell. We further investigated the apoptotic functions of membranous and soluble RCAS1 in OSCC cell lines.

## Methods

### Cell lines

Four human OSCC cell lines; HSC-2, HSC-3, SQUU-A, and SQUU-B, the uterine adenocarcinoma cell line SiSo as positive control and erythroid leukemia cell line K562 (which expresses the putative RCAS1 receptor) were used in the study. HSC-2 and HSC-3 were established from tumors of metastatic lymph nodes originating in oral squamous cell carcinomas of different patients [27]. SQUU-A and SQUU-B were established by our department from the same patient with recurrent tongue carcinoma using orthotopic implantation [28]. SQUU-B shows histologically invasive growth and has high metastatic potential (86.7% incidence of cervical lymph node metastasis), whereas SQUU-A demonstrates expansive growth and has low metastatic ability. Cells were maintained in Dulbecco’s modified Eagle’s minimum essential medium (DMEM) supplemented with 10% fetal bovine serum (FBS) and incubated at 37°C in a 5% CO_2_ atmosphere. Culture medium was changed on alternate days during the experiments. This study proposal was approved by the ethical committee of Kyushu University (No. 25–227).

### RNA extraction and reverse transcriptase-PCR

OSCC and SiSo cells were cultured in 10 cm dishes to 70–80% confluence. Total RNA was then extracted from cultured cells that were harvested with a cell scraper using Sepasol-RNA Super G (Nacalai Tesque, Kyoto, Japan) according to the manufacturer’s instructions. Primers were chosen through the GenBank sequence database (NCBI, Bethesda, MD, USA). Primer pairs for RCAS1 and glyceraldehyde-3-phosphate dehydrogenase (*GAPDH*) are listed in Table 1. All oligonucleotides used as primers were synthesized by Genenet Co., Ltd (Fukuoka, Japan). After reverse transcription with isolated mRNA, reaction products were subjected to 28 cycles of PCR for *RCAS1* and *GAPDH* at 15 seconds at 94°C for denaturation, 30 seconds at 60°C for annealing, and 60 seconds at 68°C for extension. PCR products were electrophoresed on 2% agarose gels. Bands were visualized by using ethidium bromide. Experiments were made in triplicate. Luminosity values were quantified using ImageJ software (NIH, Bethesda, MD, USA). Mean values of triplicate measurements were calculated.

**Table 1:** Primer sequence and fragment size of RCAS1

	**Forward primer**	**Reverse primer**	**Fragment size (bp)**
*RCAS1*	5′ATGGCCATCACCCAAGTTTCG-3′	5′-TTATGAAAGTTTCACACCAATT-3′	642
*GAPDH*	5′TGACCTTGCCCACAGCCTT-3′	5′-CATCACCATCTTCCAGGAGCG-3′	443

### Immunocytochemistry

OSCC and SiSo cells were separately cultured on cover glasses to 50–60% confluence. Cultured cells were fixed in 75% methanol and then incubated with anti-mouse IgM RCAS1 antibody (MBL, Nagoya, Japan) for 2 hours. The cells were then incubated with secondary antibodies conjugated with anti-mouse IgM Alexa Fluor® 488 antibodies (Invitrogen, Carisbad, CA, USA) for 1 hour., and then counterstained with Hoechst 33342 (Molecular Probes, Eugene, Oregon, USA) for 5 minutes. Fluorescently labeled cells were observed under a fluorescence microscope BZ-9000 (Keyence, Osaka, Japan).

### Flow cytometry

OSCC and SiSo cells were separately cultured in 10-cm dishes to 70–80% confluence. The cells were removed from dishes with 0.5% Trypsin/EDTA (Invitrogen) and collected by centrifugation (1200 rpm, 5 min). Cultured cells were harvested and incubated on ice for 45 min with RCAS1 antibody. After washing, the cells were incubated on ice for 45 min with anti-mouse IgM Alexa Fluor® 488 antibodies (Invitrogen), washed again and subjected to flow cytometry (FACSVerse, Becton Dickinson, San Jose, CA, USA). Isotype anti-mouse IgM antibodies (eBioscience, San Diego, CA, USA) was used as a negative control antibodies. Results were expressed as mean fluorescence intensity (MFI). Mean values of quadruplicate measurements are shown.

### Dot blotting

OSCC and SiSo cells were separately cultured in 10-cm dishes to 70–80% confluence. The culture supernatants were harvested for every 24-h incubation period from day 1 to day 4. Cell-free culture media treated under the same conditions was used as a control. To detect secreted RCAS1 in cell culture supernatants, chemiluminescent western dot blotting was performed. Briefly, each sample was applied to a nitrocellulose filter by a blotting apparatus (Rio-Rad, Richmond, CA, USA). Samples were treated with 3% hydrogen peroxide for 10 min at room temperature to remove endogenous peroxidase activity and air-dried. Next, filters were soaked in 5% nonfat milk in Tris-buffered saline-Tween 20 (TBS-T) containing 5% normal goat serum for 45 min at room temperature, then incubated with RCAS1 antibody in TBS-T containing 5% normal goat serum for 1 h at room temperature. After washing three times with TBS-T, filters were incubated for 1 h with peroxidase conjugated goat anti-mouse IgM–HRP antibodies (Santa Cruz Biotechnology, Santa Cruz, CA, USA). After washing three times with TBS-T, filters were soaked with enhanced chemiluminescence reagents (SuperSignal West Pico Chemiluminescent Substrate ThermoScientific, Rockford, IL, USA) and visualized on X-ray films.

### ELISA

OSCC and SiSo cells were cultured separately in 10-cm dishes to 70–80% confluence. Media were changed, and the culture supernatants harvested, every after 24 h for days 1–4. Cell-free culture media treated under the same conditions was used as a control. In testing for soluble RCAS1 in OSCC cell lines, RCAS1 concentrations in culture supernatant were measured in triplicate with a RCAS1 ELISA kit (Cusabio Biotech Co., LTD, Wuhan, Hubei Province, P.R. China), according to manufacturer’s instructions. Mean concentrations of quadruplicate measurements are shown.

### siRNA transfection in SQUU-B

Transfections were performed by using the X-treme- GENE siRNA transfection reagent (Roche, Mannheim, Germany), according to manufacturer’s protocol. Briefly, SQUU-B cells were added to each well of a 6-well culture plate containing complete DMEM with 10% FBS and incubated overnight for cell attachment. Unattached cells were then removed by washing and fresh DMEM medium (without antibiotics nor 10% FBS) was added. Transfection reagent was mixed with *RCAS1*-siRNA (CST, Tokyo, Japan) or negative control siRNA (Sigma-Aldrich, lrvine, CA, USA) and incubated for 15 minutes at room temperature. The resulting complex was then added to each plate. After 2 days, medium was changed to new one containing 10% FBS. The siRNA function was confirmed by RT-PCR, flow cytometry, and ELISA.

### Evaluation of apoptosis

To measure the apoptotic effect of RCAS1 as a whole in K562, OSCC cells, SiSo and the *RCAS1*-knockdown SQUU-B cells were co-cultured with K562 cells in the 6-well cell culture plate. To measure the effect of soluble RCAS1, SQUU-B cells and K562 cells were separately co-cultured in a 6-well culture plate with a cell culture insert (transparent PET membrane, 1.0-μm pore size, BD Falcon, BD Biosciences, Franklin Lakes, NJ, USA). Then, 1 × 10^5^ of each effector and K562 target cell were co-incubated in a 6 well-plate at effector/target (E/T) ratios of 1:1, 5:1, 10:1, and 20:1. To discriminate K562 cells from effector cells, K562 cells were stained by the Green fluorescence cell linker PKH67 kit (Sigma, St.Louis, MO, USA) before initiating the co-culture. Suspended cells were harvested and stained with Annexin V-PE apoptosis detection kit (eBioscience) on days 1–4 after the experiment. Flow cytometry was used to count apoptotic cells after Annexin V-PE staining on days 1–4.

### Statistical analysis

We assessed differences in RCAS1 expression and secretion, and the Annexin-V positive ratio between different cell groups with the Mann–Whitney *U-*test. *P* < 0.05 was considered to be statistically significant.

## Results

### Expression of RCAS1 mRNA and protein in OSCC cell lines

**Figure 1:**
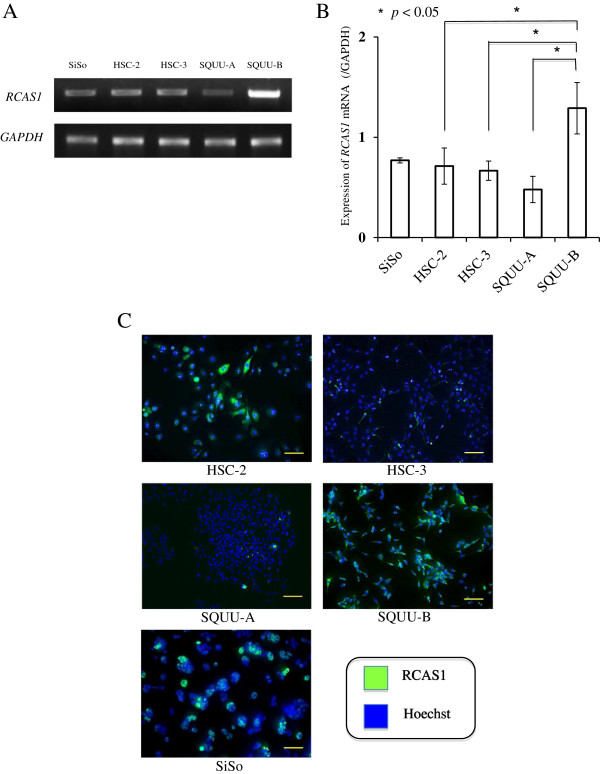
***RCAS1 *expression in OSCC cell lines (HSC-2, HSC-3, SQUU-A, SQUU-B) and SiSo (uterine adenocarcinoma cells; positive control) using RT-PCR and immunocytochemistry. A***RCAS1* mRNA was firmly detected in all OSCC cell lines. **B** Analysis for luminosity (Image J software) revealed that *RCAS1* mRNA of highly metastatic SQUU-B significantly scored higher than did the other 3 OSCC cells (*P* < 0.05; Mann–Whitney *U*-test). Mean values of triplicate measurements are shown. **C** RCAS1 protein was diffusely expressed in both cytoplasm and on membranes in all OSCC cell lines and SiSo, and was especially strong in SQUU-B cells (magnification × 10; bar: 100 μm).

RT-PCR results revealed that *RCAS1* mRNA was detected in all OSCC cell lines as in the positive control, SiSo (Figure 1A). Luminosity analysis revealed that highly metastatic SQUU-B significantly expressed more *RCAS1* mRNA than the other 3 OSCC cells (Figure 1B). Immunocytochemical results revealed that RCAS1 proteins were diffusely expressed both in cytoplasm and membrane of all OSCC cell lines as in SiSo, and was more strongly expressed in SQUU-B (Figure 1C).

### Expression of membranous RCAS1 in OSCC cell lines

**Figure 2:**
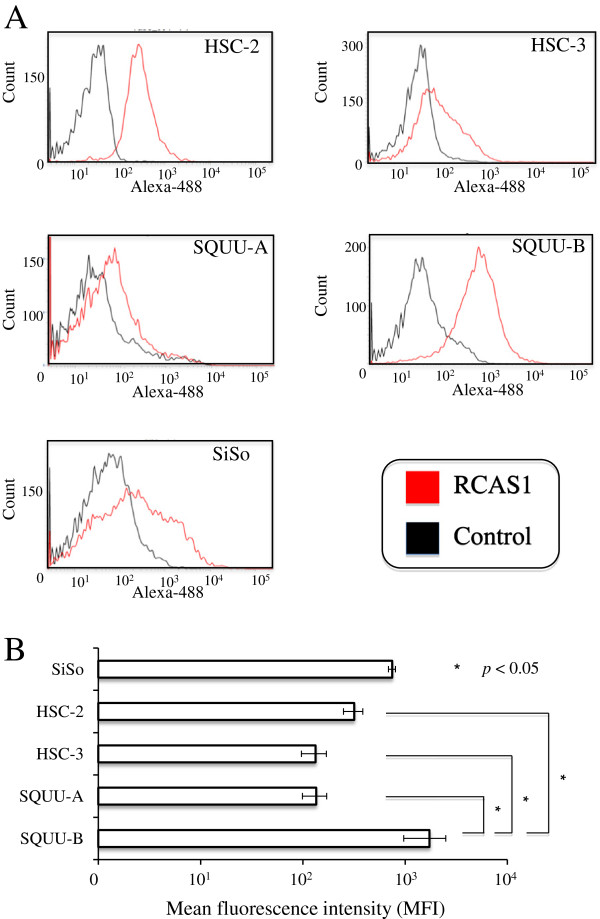
**Expression of membranous RCAS1 in OSCC cell lines using flow cytometry. A** Membranous RCAS1 was certainly expressed in all OSCC cell lines. **B** Mean fluorescence intensity (MFI) of membranous RCAS1 was significantly higher in SQUU-B than in the other 3 OSCC cell lines (*P* < 0.05; Mann–Whitney *U*-test). Mean values of quadruplicate measurements are shown.

Since RCAS1 is reportedly found in membranous and soluble patterns in several cancers, we first evaluated patterns of RCAS1 expression in OSCC cell lines. Flow cytometric analyses revealed membranous RCAS1 to be expressed in all OSCC cell lines (Figure 2A). Results were statistically compared by mean fluorescence intensity (MFI). MFI of membranous RCAS1 was significantly higher in SQUU-B (1733.8 ± 764.9) than in the other OSCC cells: HSC-2 (317.6 ± 68.2), HSC-3 (133.3 ± 36.62), and SQUU-A (135.0 ± 36.1) (*P* < 0.05; Mann–Whitney *U*-test; Figure 2B).

### Detection of soluble RCAS1 in supernatant of OSCC cells

**Figure 3:**
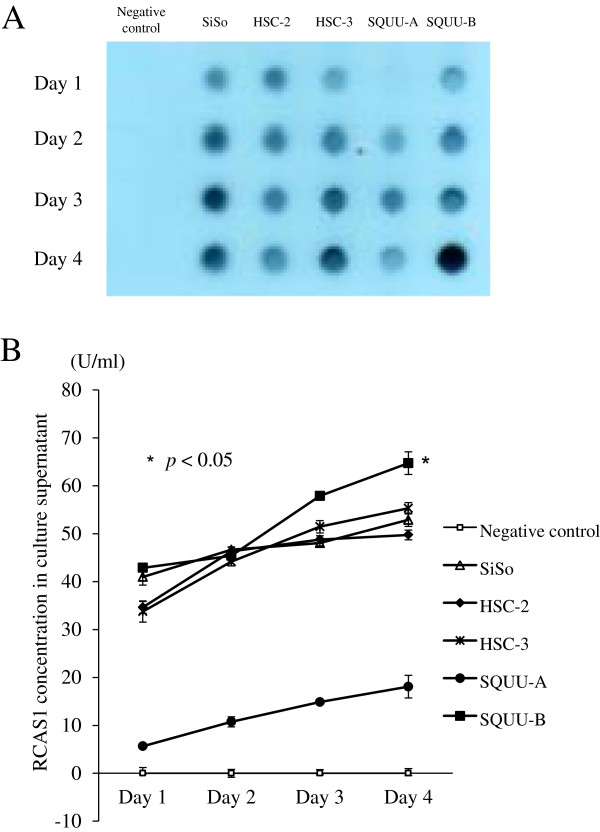
**Detection of soluble RCAS1 in OSCC cell lines using dot blotting and ELISA. A** Analyses of dot blotting revealed soluble RCAS1 in culture supernatants in all OSCC cell lines, which increased according to culture period. **B** ELISA analyses revealed significantly more soluble RCAS1 in the culture supernatant of SQUU-B than in the other 3 OSCC cell lines at day 4 (*P* < 0.05; Mann–Whitney *U*-test). Mean concentrations of quadruplicate measurements are shown.

Culture supernatants of OSCC cells were next examined for soluble RCAS1. Results of dot blotting revealed that soluble RCAS1 in culture supernatants was detected in all OSCC cell lines, which increased in amount over the culture period (Figure 3A). ELISA analyses found values of soluble RCAS1 to be 49.7 ± 1.0 U/ml for HSC-2, 55.31 ± 1.2 U/ml for HSC-3, 18.1 ± 2.4 U/ml for SQUU-A, and 64.7 ± 0.5 U/ml for SQUU-B by day 4, while were 34.6 ± 1.21 U/ml for HSC-2, 33.8 ± 1.2 U/ml for HSC-3, 6.7 ± 0.4 U/ml for SQUU-A, and 41.9 ± 0.3 U/ml for SQUU-B by day 1. The SQUU-B supernatant significantly carried more soluble RCAS1 than the other OSCC cell lines by day 4 (*P* < 0.05; Mann–Whitney *U*-test, Figure 3B).

### Apoptosis of K562 cells induced by RCAS1 from OSCC cells

**Figure 4:**
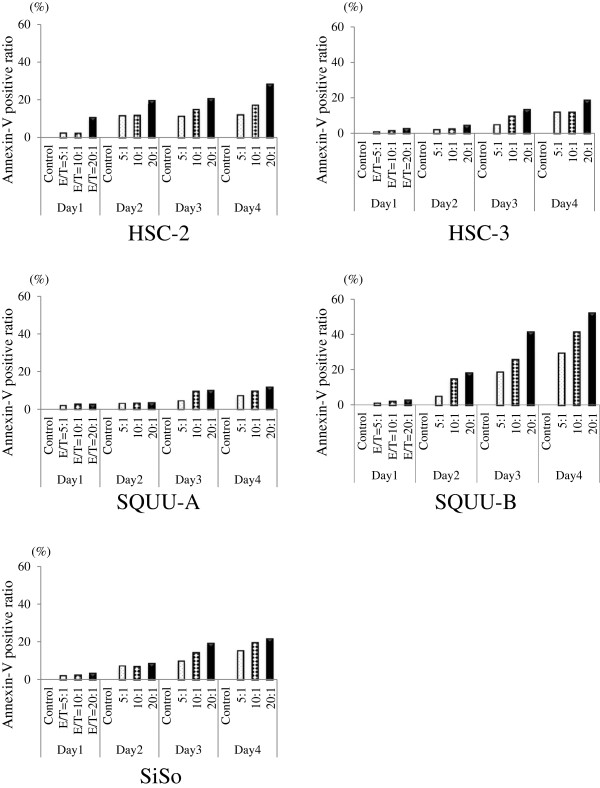
**Representative results for Annxin-V positive ratio of K562 cultured with OSCC cell lines.** Apoptosis rates for K562 cells gradually increased by E/T ratios and culture periods in all conditions of co-culture for each OSCC cell lines. Apoptosis of K562 was more induced in the co-culture with SQUU-B cells than with the other 3 OSCC cell lines.

RCAS1-induced apoptosis in K562 cells was analyzed using a co-culture system with OSCC cells. Each OSCC cell line induced apoptosis in K562 cells. The Annexin V positive ratio of K562 increased with E/T ratios and culture periods under all co-culture conditions with each OSCC cell line. As representative results, the ratio increased in an E/T ratio-dependent fashion by co-culture day 4; 28.4% with HSC-2, 16.6% with HSC-3, 10.8% with SQUU-A, and 52.5% with SQUU-B at a 20:1 E/T ratio. Apoptosis of K562 cells were more strongly induced by co-culture with SQUU-B cells than the other 3 OSCC cell lines (Figure 4).

**Figure 5:**
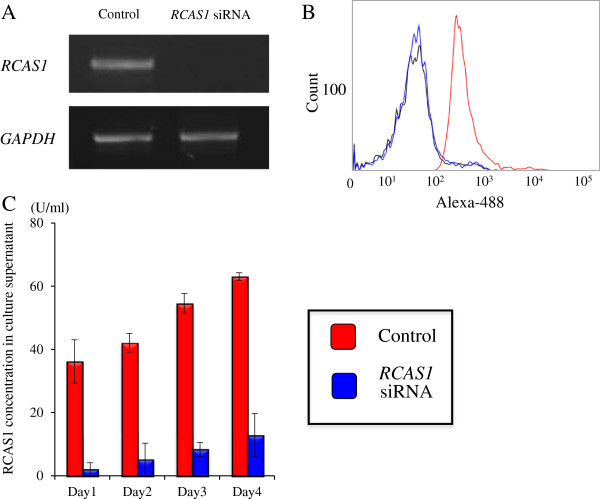
***RCAS1 *siRNA transfection in SQUU-B. A** RT-PCR analyses revealed that *RCAS1* siRNA successfully reduced *RCAS1* mRNA levels in SQUU-B. **B** Flow cytometric analyses revealed that *RCAS1* siRNA certainly down-regulated the expression of membranous RCAS1 in SQUU-B. **C** ELISA analyses revealed that RCAS1 siRNA definitely down-regulated the soluble RCAS1 in SQUU-B through the culture periods. Mean concentrations of quadruplicate measurements are shown.

**Figure 6:**
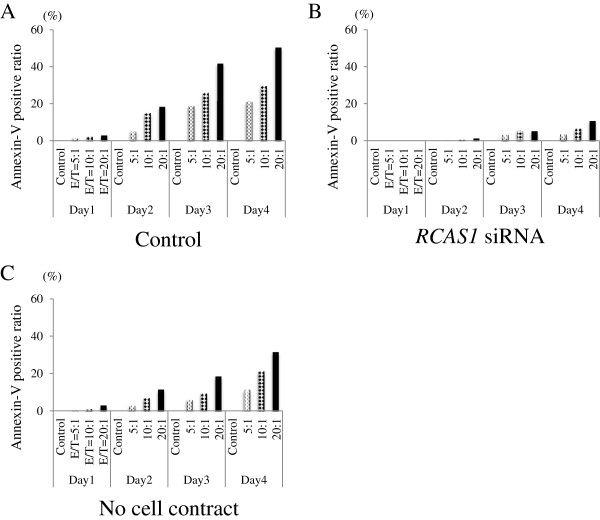
**Representative results for Annxin-V positive ratio of K562 via membranous and soluble RCAS1 in SQUU-B. A** Annexin-V positive ratio of K562 cultured with SQUU-B (positive control). Apoptotic rates for K562 cells gradually increased according to E/T ratios and culture periods. **B** With the *RCAS1 si*RNA transfection, apoptosis of K562 was remarkably inhibited. **C** In the co-culturing of K562 cells with SQUU-B without cell contact (using a cell culture insert), K562 apoptosis was still induced.

Next, the same assay was conducted under different conditions of co-culture with *RCAS1* knockdown SQUU-B cells and no-contact co-culture using a cell culture insert. First, results of RT-PCR, flow cytometry, and ELISA confirmed that the *RCAS1* siRNA transfection was successful and the *RCAS1* was silenced (Figure 5A–C). When K562 cells were cultured with SQUU-B as positive control, the Annexin-V positive ratios for K562 were 1.5% at a 5:1 E/T ratio, 2.2% at 10:1 and 3.0% at 20:1 by day 1; 5.22% at 5:1, 15.0% at 10:1, and 18.4% at 20:1 by day 2; 19.2% at 5:1, 25.9% at 10:1 and 41.7% at 20:1 by day 3; and 21.3% at 5:1, 29.7% at 10:1 and 50.5% at 20:1 by day 4. When K562 cells were cultured with *RCAS1* knockdown SQUU-B cells, Annexin-V positive ratio of K562 were 0.2% at 5:1, 0.5% at 10:1, and 0.6% at 20:1 by day 1; 0.3% at 5:1, 0.7% at 10:1, and 1.3% at 20:1 by day 2; 3.2% at 5:1, 5.6% at 10:1, and 5.1% at 20:1 by day 3; and 3.4% at 5:1, 6.7% at 10:1 and 10.8% at 20:1 by day 4. When co-cultured with the *RCAS1-*knockdown cells, 41.7% of the Annexin-V positive cells were inhibited at a 20:1 E/T ratio by day 4. Apoptosis of K562 cells was remarkably inhibited when co-cultured with *RCAS1* knockdown SQUU-B cells through the culture periods (Figure 6).

Even when K562 cells were cultured with SQUU-B without cell contact, using the cell culture insert, the K562 Annexin-V positive ratio measured by flow cytometry showed apoptosis increased due to culture periods and E/T ratios: 0.7% at 5:1, 1.0% at 10:1, and 3.8% at 20:1 by day 1; 3.9% at 5:1, 7.0% at 10:1, and 11.4% at 20:1 by day 2; 6.3% at 5:1, 9.4% at 10:1, and 18.5% at 20:1 by day 3; 11.4% at 5:1, 21.3% at 10:1, 31.5% at 20:1 by day 4. Thus, apoptosis of K562 cells was induced when cultured with only soluble RCAS1 (Figure 6).

## Discussion

To our knowledge, this is the first report to find membranous and soluble RCAS1 in OSCC. Expression of *RCAS1* mRNA in OSCC cells accords with that in 10 kinds of breast cancer cells [29] and 4 kinds of cholangiocarcinoma cells [30]. Moreover, expression of RCAS1 protein in OSCC cells accords with that in 4 kinds of breast cancer cells [31]. These consistencies encouraged us to further investigate its expression patterns, as immunocytochemical analysis individual cannot distinguish between membranous and soluble RCAS1.

Results of flow cytometry for membranous RCAS1 in OSCC cells are congruent with those for SiSo and TMCC (uterine cervical cancer), MH and Kuramochi (ovarian mucinous cancer), KF, HRA (ovarian serous cancer), and MCF-7 (breast cancer) [2,32]. Results of the dot blotting and ELISA suggest that the soluble RCAS1 could be definitely detected in all of OSCC cells and its concentrations were time-dependent. These results align with findings that soluble RCAS1 in serum is significantly increased in colon cancer patients compared with that in healthy individuals; higher concentration of soluble RCAS1 is significantly associated with advanced Dukes’ stage and high histopathological tumor grade; colon cancer patients with the higher concentration had significantly shorter overall survival time; and soluble RCAS1 is an independent prognostic factor for this malignancy [33,34].

Interestingly, the significant luminosity of *RCAS1* mRNA and the significant MFI value of membranous RCAS1 in SQUU-B suggest that RCAS1 expression is associated with its high metastatic potential, as does its significant increase in soluble RCAS1 levels by day 4. Therefore, highly metastatic OSCC cells are likely to express RCAS1 as membranous or soluble proteins. In cervical and ovarian cancers, the number of cells that express mesenchymal marker vimentin is inversely related to RCAS1 expression, as remodeling of stromal tissue by vimentin results in tumor invasion and metastasis [35]. Moreover, enhanced RCAS1 expression in African Green Monkey kidney fibroblast cell lines COS-7 following introduction of the *RCAS1* gene significantly promotes *in vivo* tumor growth via increase of vascular endothelial growth factor [36], which implies that RCAS1 affects tumor progression through lymph nodal metastasis. Combined with the significant expression of RCAS1 in SQUU-B with high metastatic potential, those findings suggest that RCAS1 may play a pivotal role in tumor progression by lymph nodal metastasis.

Since both results of apoptotic cell detection by staining of Annexin V and propidium iodide were synchronized *in vitro*[21] and we could confirm that apoptosis of tumor-infiltrating lymphocytes was found by TUNNEL staining *in vivo*[25], the staining of Annexin V individual was used for the detection in this study. Results of flow cytometry indicate that apoptosis of K562 cells is markedly induced in the co-culture system with each OSCC cells, and this incidence was dependent on RCAS1 concentration and culture periods. These results do not align with reports that K562 apoptosis was not induced in co-culture with MCF-7 breast cancer cells [22]. Therefore, to assess the extent of this apoptotic ability from soluble RCAS1, we used *RCAS1* siRNA transfection to create an *RCAS1*-silenced SQUU-B with high metastatic potential. We then co-cultured K562 cells in medium shared with either *RCAS1*-silenced or SQUU-B without direct cell contact. By day 4 at a 20:1 E/T ratio, the Annexin-V positive ratio was 31.5% for K562 cells cultured with SQUU-B without direct contact, and 50.5% in K562 cells cultured in contact with SQUU-B, which suggests that 31.5% was induced by soluble RCAS1 versus 19.0% by membranous RCAS1. Since membranous/soluble RCAS1 value from SQUU-B was absolutely higher than that from positive control SiSo and SQUU-B is specific with high metastatic potential, it might be possible that even membranous RCAS1 had the apoptotic ability in this study. Moreover, soluble RCAS1 apparently affects apoptotic ability of OSCC cell more than membranous RCAS1. Though apoptotic ability of membranous and soluble RCAS1 in K562 was merely analyzed in this study, it has been reported that the cell growth of K562 was inhibited, after the culture for 72 hours with the serum including soluble RCAS1 of more than 10 U/ml from patients with uterine cancer [23]. In addition, it has been also reported that the apoptosis of activated T cells by IL-2 was markedly induced after culture for 72 hours with recombinant RCAS1-glutathione S-transferase fusion proteins, while the apoptosis of CD3-positive (non-activated) T cells was not [21]. Combined these results with our findings, though immune system of patient initially finds cancer occurred via T cell recognition and TILs attack the cancer to prevent from the growth and the invasion, RCAS1 could finally induce apoptosis of TILs such as T cells in patients with OSCC. We previously reported that patients with RCAS1 positive OSCCs have significantly higher percentages of TUNEL positive TILs than do patients with RCAS1 negative OSCCs [25]. These results accord with reports that TIL apoptosis rates in colorectal [10] and gastric carcinomas [13] were significantly higher in RCAS1 positive tissues than in RCAS1 negative tissues. Therefore, RCAS1-induced apoptosis of TILs in patients with OSCC can lead to escape of tumor cells from immune surveillance and contribute to tumor growth *in vivo*.

RCAS1 could be defined as tumor-associated antigen in OSCC as well as in other cancers. There could be clarified to be two patterns of RCAS1 expression in OSCC and the apoptotic ability of soluble RCAS1 could impact on the total ability of tumor cell more than that of membranous one like other cancers. In addition, the possibility of RCAS1 has been proposed as a biomarker of ovarian and uterine cancer [3,23]. Thus, detection of soluble RCAS1 in serum of patients with OSCC might be available as a biomarker of OSCC to facilitate diagnosis, therapeutic strategies and disease monitoring, as well as improving insight into the disease state. If so, RCAS1 could be applicable for OSCC treatment as a targeted molecule. Several strategies for RCAS1 could be considered such as modulation of RCAS1 with siRNA or inhibition of RCAS1 with monoclonal antibody, in order to suppress its expression and function. The identification of useful biomarkers is crucial and recent advances in biomarker discovery have raised new opportunities in the emerging fields of personalized and predictive medicine. Further clinical studies are warranted to investigate soluble RCAS1 in serum of patients with OSCC and to clarify the association of RCAS1 expression with clinico-pathological parameters.

## Conclusion

RCAS1 has an apoptotic function via membranous and soluble expression in OSCC cells. Moreover, soluble RCAS1 could extremely impact on the ability, compared to membranous one. RCAS1 may thus affect tumor escape from immune surveillance in OSCC by inducing apoptosis.

## Abbreviations

DMEM: Dulbecco’s modified Eagle’s minimum essential medium; E/T: Effector/target ratio; ELISA: Enzyme-linked immunosorbent assay; FBS: Fetal bovine serum; GAPDH: Glyceraldehyde-3-phosphate dehydrogenase; MFI: Mean fluorescence intensity; OSCC: Oral squamous cell carcinoma; RCAS1: Receptor-binding cancer antigen expressed on SiSo cell; TBS-T: Tris-buffered saline-Tween 2; TILs: Tumor infiltrating lymphocytes.

## Competing interests

All authors have no financial or personal relationships with other people or organisations that could inappropriately influence (bias) their work.

## Authors’ contributions

HT carried out mainly the all experiments and described the manuscript. TT especially designed RT PCR, immunocytochemistry, and siRNA transfection and described mainly the manuscript. KS especially designed flow cytometry, dot blotting, and ELISA, and provided materials. RK participated in statistical analyses. MS provided materials and participated in the study design. SK participated in the study design and helped to draft this manuscript. RM participated in the establishment of OSCC cell lines and siRNA transfection. YG participated in the establishment of OSCC cell lines, RT PCR, and immunocytochemistry. SN helped to draft this manuscript. All authors have revised this manuscript and agreed for submission.
